# COVID-19 and the Additional Radiological Risk during the Lockdown Period in the Province of Naples City (South Italy)

**DOI:** 10.3390/life12020246

**Published:** 2022-02-07

**Authors:** Giuseppe La Verde, Valeria Artiola, Marco La Commara, Vittoria D’Avino, Leopoldo Angrisani, Giuseppe Sabatino, Mariagabriella Pugliese

**Affiliations:** 1 National Institute for Nuclear Physics (INFN), Via Cinthia ed. 6, 80126 Naples, Italy; lacommar@na.infn.it (M.L.C.); vdavino@na.infn.it (V.D.); pugliese@na.infn.it (M.P.); 2 Department of Physics “E. Pancini”, University of Naples Federico II, Via Cinthia ed. 6, 80126 Naples, Italy; 3 Centre for Advanced Metrology and Technological Services (CeSMA), University of Naples Federico II, Corso Nicolangelo Protopisani, 80146 Naples, Italy; v.artiola@studenti.unina.it (V.A.); giuseppe.sabatino@unina.it (G.S.); 4 Department of Pharmacy, University of Naples Federico II, Via Domenico Montesano, 49, 80131 Naples, Italy; 5 Department of Information Technology and Electrical Engineering, University of Naples Federico II, Via Claudio, 21, 80125 Naples, Italy; leopoldo.angrisani@unina.it

**Keywords:** COVID-19, lockdown, radon, indoor air quality, radiological risk

## Abstract

The lockdown restrictions, as a first solution to contain the spread of the COVID-19 pandemic, have affected everyone’s life and habits, including the time spent at home. The latter factor has drawn attention to indoor air quality and the impact on human health, particularly for chemical pollutants. This study investigated how the increasing time indoor influenced exposure to natural radioactive substances, such as radon gas. To calculate the radiological risk, we considered the most consolidated indices used for radiation protection: annual effective dose, excess lifetime cancer risk, and the lung cancer case. Furthermore, two different exposure times were considered: pre-lockdown and post-lockdown. The lockdown increased the indoor exposure time by 4% and, consequently, the radiological risk factors by 9%. Furthermore, the reference value of 300 Bq/m^3^, considered acceptable for human radiation protection, may need to be lowered further in the case of conditions similar to those of the lockdown period.

## 1. Introduction

The spread of the COVID-19 disease [1] has imposed several changes in the habits of the entire world population, affecting, above all, both the economic and social spheres [2]. The World Health Organisation (WHO) recommended a lockdown to contain the disease [3], a suggestion that was approved by several nations, including Italy [4,5]. This first containment action was due to an initial lack of knowledge of the biology and of pathway transmission of the virus, also reflecting the evolution of the risk perception [6]. The initial assumption was that the virus was airborne and therefore linked to environmental conditions [7], and thus people who were in lockdown often preferred to stay indoors, avoiding air changes from the outside. Therefore, as a result of the lockdown and the stopping of most production and transport activities, the reduction of emissions improved the quality of the outdoor air [8,9,10,11,12,13,14,15,16] and water [17,18]. The study of indoor air quality (IAQ), which has increasingly attracted scientific interest due to its close correlation with human health [19,20,21,22,23,24], has also been deepened in relation to this pandemic [25,26,27,28,29,30,31], uniquely highlighting the worsening of IAQ. The results confirm that the lockdown has increased the concentration of indoor pollutants, especially chemicals such as CO_2_, PM_2.5_, PM_10_, NO_2_, SO_2_, and volatile organic compounds (VOCs); otherwise, to date, there have been few evaluations on exposure to radioactive substances normally found indoors [32].

From a radiological point of view, the direct consequence of the lockdown measure is the increase in time spent indoors by the population, which has effectively increased the exposure to radioactive elements naturally produced by the Earth’s crust and/or building materials. Among these kinds of pollutants, probably the most important one is radon-222 (^222^Rn). ^222^Rn is a noble gas generated by the radioactive decay of ^238^U and characterised by a half-life of 3.8 days. The health risk of this gas is given by its progeny (^218^Po, ^214^Pb, ^214^Bi, ^214^Po), which can cause serious DNA damage due to the emission of alpha particles [33,34,35,36,37]. In fact, alpha particles have a high LET, and thus they have the ability to deposit high quantities of energy in small distances [38]. Because of its potential adverse effects, ^222^Rn has been classified by the WHO as a health hazard and the primary cause of lung cancer in non-smokers [39].

Due to its relevance, there are many studies regarding its distribution, the processes that favour indoor accumulation, and the impact on health. For example, our research group has focused its attention on the analysis of the concentration distribution of natural radionuclides, especially radon gas in the territory of South Italy [40,41,42,43,44,45,46,47,48,49,50,51,52,53,54,55,56,57,58,59], as well as on the investigations on several aspects of both natural and artificial radioactivity concerning in particular the structure, the decay modes, and the optical model calculations of light radioactive isotopes [60,61,62,63,64,65].

As previously mentioned, the inhalation of radon gas can lead to several forms of damage to one’s health [66,67]. The International Commission on Radiological Protection (ICRP) has published several fundamental recommendations to ensure the safety of both workers and the general population from exposure to ^222^Rn [68,69,70,71,72,73]. Furthermore, in 2013, with the introduction of the European Directive EURATOM 59/2013 [74], the evaluation of indoor air quality was required from the UE nations for the first time. In particular, the directive states that the reference level for ^222^Rn activity concentration in the air is equal to 300 Bq/m^3^ both for workplace (art. 54) and for other environments, such as homes (art. 74). In 2020, Italy implemented the directive with the Italian Decree n.101 [75].

There are different parameters that are used to evaluate radon-correlated health risk. Among these, those worth mentioning are the annual effective dose (AED), excess lifetime cancer risk (ELCR), and the lung cancer case (LCC). These values have been proposed by UNSCEAR, who also offer the method for their calculation [76].

In this study, the assessment of the health risk due to inhalation of ^222^Rn during the lockdown was conducted in various countries of Naples, found in the Campania region (South Italy), using radon gas activity concentration values obtained in previous territorial characterisation surveys, constituting our database.

## 2. Methods

### 2.1. Exposure Time

Exposure time is one of the parameters used to estimate the radiological risk of radon gas. The ICRP 93 report [35] states that people spend, on average, 7000 h/year at home and 2000 h/year at the workplace. However, with the forced isolation measures imposed to avoid the spread of COVID-19, the hours spent at home considerably increased. In Italy, since the lockdown lasted for 2 months, the time spent at home was equal to 7293 h/year. Hence, in this study, the values obtained through the evaluation of the exposure risk due to ^222^Rn in normal conditions were compared with the ones calculated considering 2 months of lockdown.

### 2.2. Study Area

The study of the radiological risk was conducted in 19 of the 92 municipalities of the province of Naples, located in the Campania region (South Italy), for a total of 496 measurements of activity concentration of ^222^Rn. The sampling area is characterised by very peculiar geological settings [77]. For instance, the traditional construction system used in this region involves the use of several stones of volcanic origins, such as tuff, which, as it is known, can influence a greater accumulation of radon indoors. In fact, radon mean activity concentration calculated in this study area, equal to 107 ± 30 Bq/m^3^, is higher than the national average level (≈75 Bq/m^3^) [78].

### 2.3. Health Risk Factors

As previously mentioned, several factors are considered fundamental in the estimation of health risks linked to air quality. In this study, the analyses were conducted through the calculation of

- The annual effective dose;
- The excess lifetime cancer risk;
- The lung cancer case.

#### 2.3.1. Annual Effective Dose

The AED, a parameter used to define the dose absorbed by the general population subjected to gas radon exposure in a year, was calculated using the Equation (1) found in the UNSCEAR 2000 report [76]:

AED = C_Rn_ × C_inh_(Rn) × F_eq_ × F_occ_ × T (1)

where C_Rn_ represents radon concentration, C_inh_(Rn), which is equal to 9 nSv h Bq m^−3^ [76], being the effective dose coefficient for indoor radon exposure; F_eq_ is the equilibrium factor equal to 0.4; F_occ_ is the occupation factor, equal to 1 for the analyses post-lockdown period and 0.8 for the pre-lockdown period; and T is the exposure time, expressed in hours.

#### 2.3.2. Excessive Lifetime Cancer Risk

The ELCR indicates the likelihood of developing cancer later in life due to exposure to ionising radiation. The ELCR is given by the following Equation (2):

ELCR = AED × DL × RF(2)

where DL is the average lifespan of the population and RF is the risk of fatal cancer in sieverts. In particular, the former factor for inhabitants of Naples was equal to 77.2 years for men and 82.2 for women [79]. According to the ICRP 2007 report [69], the RF factor is equal to 5.5 × 10^−2^ Sv^−1^.

#### 2.3.3. Lung Cancer Case

The LCC is a factor calculated per year per million people and it considers the probability for people to develop lung cancer due to exposure to radiation. The LCC is calculated through employing the following Equation (3):

LCC = AED × 18 × 10^−6^ (3)

where 18 × 10^−6^ indicates the probability of developing cancer [80].

## 3. Results and Discussion

**Figure 1:**
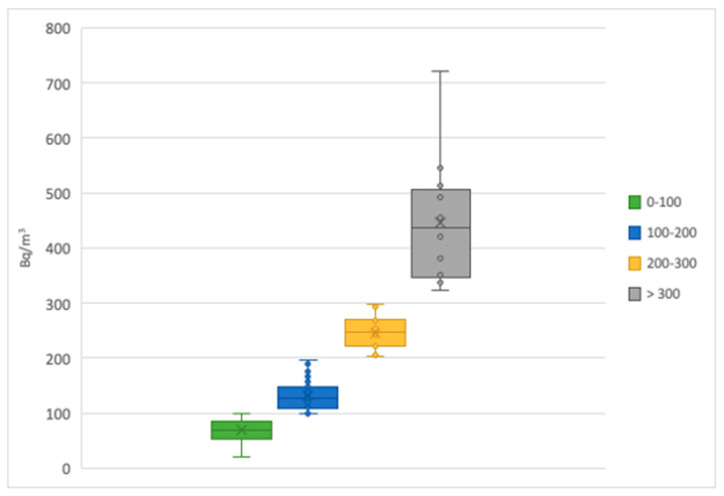
Variability of radon activity concentrations.

The annual average ^222^Rn activity concentrations were divided into four groups on the basis of the mean concentration in order to illustrate the distribution of the measurements. The obtained results are listed in Table 1, and the variability of the concentration values are shown in Figure 1.

Most of the analysed houses were characterised by a radon activity concentration lower than 100 Bq/m^3^, while only 12 of them showed a ^222^Rn concentration higher than the reference level of 300 Bq/m^3^ [73,75]. All the health risk factors were evaluated in two different conditions: pre- and post-lockdown. For reference values, ICRP 137 data were used [73]. To evaluate radon effective dose, we used the mean activity concentration values, taking into account the pre- and post-lockdown periods.

### 3.1. Annual Effective Dose

To evaluate the AED, we used Equation (1). The results are listed in Table 2:

The comparison between the AED values obtained using the different exposure times shows that the results relative to the post-lockdown period were 9% higher than the ones obtained when considering the exposure time of 7000 h/year indicated in [68], an increment observed for all the following parameters.

For the ranges 0–100 Bq/m^3^ and 100–200 Bq/m^3^, the AED values were well below the reference value of 6.05 mSv/year [73], as it was expected that AED would be greater than the reference for concentrations in the range >300 Bq/m^3^.

On the contrary, the AEDs calculated for the range 200–300 Bq/m^3^ were below the reference for both the pre- and post-lockdown periods when calculated with the average values (Table 2); however, analysing in detail each measurement point and the corresponding AEDs, we found that 9 homes out of 25 (36% of the total) had radon activity concentration values ≥274 Bq/m^3^, representing an interesting and critical value at the same time. In fact, as shown in Table 3, 2 months of lockdown influenced the exposure and consequently the AED, which from values below 6.05 mSv/year all became worryingly greater than the reference value.

### 3.2. Excess Lifetime Cancer Risk

The ELCR index is a fundamental tool used in radiobiology to estimate the health risk due to indoor radon. In particular, this parameter offers a clear view of the great impact of the lockdown measure, leading to a significant increase in the probability of developing cancer.

The ELCR values calculated with Equation (2), for both the pre- and post-lockdown conditions, are listed in Table 4.

For ELCR, the trend did not change, as in precedence, and therefore we report the details of the cases in which the impact of the lockdown was evident (Table 5), exceeding the reference level of 2.66 × 10^−2^ [73]. This trend was even more sensational when ELCR was analysed by gender.

Considering the ELCR calculated for a general average age 79.7 years and for the post-lockdown period, we found that the radon activity concentration value corresponding to an ELCR greater than the reference of 2.66 × 10^−2^ was 276 ± 77 Bq/m^3^ (ELCR = 2.67 × 10^−2^).

On the contrary, making an analysis by gender and therefore by different life expectancies, we found that for men, the post-lockdown ELCR varied from 2.52 × 10^−2^ to 2.80 × 10^−2^, and the critical value of radon concentration, corresponding to a value higher than the reference level, was 294 ± 82 Bq/m^3^ (building #5). The post-lockdown scenario was different for women, for whom ELCR varied from 2.68 × 10^−2^ to 2.98 × 10^−2^, and the critical radon concentration value corresponding to an ELCR higher than the reference was 269 ± 75 Bq/m^3^ (building #10).

### 3.3. Lung Cancer Case

As previously mentioned, the LCC per million people indicates the number of individuals affected by lung cancer in a year, and it was evaluated using Equation (3). As was the case with the other parameters analysed previously, the LCC strongly depended on the value of the exposure time. The results pre- and post-pandemic are indicated in Table 6.

The results show that, while maintaining a 9% increase due to the lockdown period, for radon activity concentrations < 300 Bq/m^3^, the LLC was <108.86, the reference value for ICRP [73]. These data must be carefully considered since lung cancer is characterised by a very high incidence and mortality rate [81]. According to the Associazione Italiana Oncologia Medica (AIOM), in Italy, the trend in incidence is different between the male gender, for which a significant reduction was observed (−6.5% compared to 2019), and the female gender, for which there was a sharp increase (+2.5%). Moreover, in 2020, approximately 41,000 new diagnoses were expected [82]. Therefore, LCC is, without a doubt, a fundamental tool from the radiobiological standpoint.

## 4. Conclusions

As previously mentioned, radon gas is one of the most dangerous indoor air pollutants, having a radiological effect on humans. There are several parameters used to analyse the radiobiological risk linked to this gas, most of them dependant on the exposure time. Therefore, the aim of this study was to evaluate the variation of these parameters due to the lockdown measure imposed in 2020. In fact, the spread of COVID-19 has led to forced isolation necessary to limit the infection, effectively increasing the time spent indoors. As a direct consequence, there has been an augmentation in the exposure time to indoor pollutants, including radon gas.

This study was conducted using a total of 496 ^222^Rn activity concentration measurements carried out in the Neapolitan area (South Italy). The concentration activity values were divided into four ranges of values, and the analysed indexes were as follows: the AED, EDL, ELCR, and LCC. All of them were estimated in pre-lockdown, and post-lockdown conditions were affected by the increase in indoor exposure time. Furthermore, some values defined as critical were also compared with the reference levels reported in ICRP 137, in the European Directive 59/2013 EURATOM, and in the national legislation [73,74,75]. The case of the 200–300 Bq/m^3^ range was very interesting, for which the influence of the lockdown, which can be translated into an increase in indoor time, also caused the reference values for radon activity concentration to be exceeded, which are not normally considered. Thus, the results suggest that the new reference limit for an exposure time similar to that caused by the lockdown is no longer 300 Bq/m^3^, but 274 Bq/m^3^, as demonstrated by the results obtained for AED. Another interesting aspect is ELCR analysed by gender. In the pre-lockdown period, no radon activity concentration value between 269 and 300 Bq/m^3^ corresponded to an ELRC greater than the reference of 2.66 × 10^−2^. On the contrary, in the post-lockdown period, the ELRC reference value was exceeded at radon activity concentrations of 293 ± 82 Bq/m^3^ for men and, even more surprisingly, of 269 ± 75 Bq/m^3^ for women. The LCC per million people, despite having been influenced by the post-lockdown period, was consistent with the reference values and therefore greater than 108.86 for values greater than 300 Bq/m^3^. In the next few years, it would be interesting to correlate the cases recorded and reported in national databases with those hypothesised in this study to understand if the lockdown had a real impact from an epidemiological point of view.

Since the spread of COVID-19 has yet to be stopped, in the event of another lockdown or a similar scenario, the results reported in this study and the new knowledge acquired on the mechanisms of spread of the virus suggest increasing the air changes in the room to prevent the accumulation of radon gas. Remedial actions to reduce radon activity concentration, in fact, are widely known [83], and among these, the cheapest and the one whose effectiveness has also been measured by our group [84] is passive ventilation: a greater exchange of air between the inside and outside by keeping windows and doors open, also producing an overall improvement in IAQ.

## Figures and Tables

**Table 1:** Values of radon activity concentration for each group.

^222^Rn Activity Concentration (Bq/m^3^)	Minimum Value (Bq/m^3^)	Mean Value (Bq/m^3^)	Maximum Value (Bq/m^3^)	N° of Houses
0–100	21 ± 6	54 ± 1	100 ± 28	304
100–200	100 ± 28	123 ± 3	197 ± 55	155
200–300	204 ± 57	240 ± 14	299 ± 84	25
>300	323 ± 90	378 ± 32	722 ± 202	12

**Table 2:** AED values before and after the lockdown period for mean value of each radon range.

^222^Rn Activity Concentration (Bq/m^3^)	AED Pre-Lockdown (mSv/Year)	AED Post-Lockdown (mSv/Year)
0–100	1.09	1.19
100–200	2.48	2.72
200–300	4.83	5.29
>300	7.61	8.33

**Table 3:** Detail of critical AED values before and after the lockdown period for building ≥274 Bq/m^3^ in the range 200–300 Bq/m^3^.

Building	^222^Rn Activity± Concentration (Bq/m^3^)	AED Pre-Lockdown (mSv/Year)	AED Post-Lockdown (mSv/Year)
#1	275 ± 77	5.54	6.07
#2	299 ± 84	6.03	6.60
#3	276 ± 77	5.56	6.08
#4	293 ± 82	5.90	6.46
#5	294 ± 82	5.93	6.49
#6	276 ± 77	5.56	6.09
#7	278 ± 77	5.60	6.13
#8	281 ± 79	5.66	6.20
#9	277 ± 78	5.58	6.11

**Table 4:** ELCR values before and after the lockdown measure for mean value of each concentration range.

^222^Rn Activity Concentration (Bq/m^3^)	ELCR Pre-Lockdown	EDL Post-Lockdown
0–100	4.79 × 10^−3^	5.24 × 10^−3^
100–200	1.09 × 10^−2^	1.19 × 10^−2^
200–300	2.13 × 10^−2^	2.33 × 10^−2^
>300	3.35 × 10^−2^	3.66 × 10^−3^

**Table 5:** Details of critical ELCR values before and after the lockdown period for buildings in the range of 200–300 Bq/m^3^.

Building	^222^Rn Activity Concentration (Bq/m^3^)	ELCR Pre-Lockdown			ELCR Post-Lockdown		
		General Average 79.7 Years	Average Men 77.2 Years	Average Women 82.2 Years	General Average 79.7 Years	Average Men 77.2 Years	Average Women 82.2 Years
#1	275 ± 77	2.43 × 10^−2^	2.35 × 10^−2^	2.51 × 10^−2^	2.66 × 10^−2^	2.58 × 10^−2^	2.74 × 10^−2^
#2	299 ± 84	2.64 × 10^−2^	2.56 × 10^−2^	2.73 × 10^−2^	2.89 × 10^−2^	2.80 × 10^−2^	2.98 × 10^−2^
#3	276 ± 77	2.44 × 10^−2^	2.36 × 10^−2^	2.51 × 10^−2^	2.66 × 10^−2^	2.58 × 10^−2^	2.75 × 10^−2^
#4	293 ± 82	2.59 × 10^−2^	2.51 × 10^−2^	2.67 × 10^−2^	2.83 × 10^−2^	2.74 × 10^−2^	2.92 × 10^−2^
#5	294 ± 82	2.60 × 10^−2^	2.52 × 10^−2^	2.68 × 10^−2^	2.84 × 10^−2^	2.75 × 10^−2^	2.93 × 10^−2^
#6	276 ± 77	2.44 × 10^−2^	2.36 × 10^−2^	2.52 × 10^−2^	2.67 × 10^−2^	2.58 × 10^−2^	2.75 × 10^−2^
#7	278 ± 77	2.46 × 10^−2^	2.38 × 10^−2^	2.53 × 10^−2^	2.69 × 10^−2^	2.60 × 10^−2^	2.77 × 10^−2^
#8	281 ± 79	2.48 × 10^−2^	2.41 × 10^−2^	2.56 × 10^−2^	2.72 × 10^−2^	2.63 × 10^−2^	2.80 × 10^−2^
#9	277 ± 78	2.45 × 10^−2^	2.37 × 10^−2^	2.52 × 10^−2^	2.68 × 10^−2^	2.59 × 10^−2^	2.76 × 10^−2^
#10	269 ± 75	2.37 × 10^−2^	2.30 × 10^−2^	2.45 × 10^−2^	2.60 × 10^−2^	2.52 × 10^−2^	2.68 × 10^−2^

**Table 6:** LCC values for each radon activity concentration range before and after the lockdown period.

^222^Rn Activity Concentration (Bq/m^3^)	LCC × 10^−6^ Pre-Lockdown	LCC × 10^−6^ Post-Lockdown
0–100	19.59	21.43
100–200	44.68	48.87
200–300	86.99	95.17
>300	137.04	149.92

## Data Availability

Data contained within the article.
